# Tendon Stem/Progenitor Cells and Their Interactions with Extracellular Matrix and Mechanical Loading

**DOI:** 10.1155/2019/3674647

**Published:** 2019-10-13

**Authors:** Chuanxin Zhang, Jun Zhu, Yiqin Zhou, Bhavani P. Thampatty, James H-C. Wang

**Affiliations:** ^1^Joint Surgery and Sports Medicine Department, Shanghai Changzheng Hospital, Second Military Medical University, Shanghai, China; ^2^MechanoBiology Laboratory, Departments of Orthopaedic Surgery, Bioengineering, and Physical Medicine and Rehabilitation, University of Pittsburgh, Pittsburgh, Pennsylvania, USA

## Abstract

Tendons are unique connective tissues in the sense that their biological properties are largely determined by their tendon-specific stem cells, extracellular matrix (ECM) surrounding the stem cells, mechanical loading conditions placed on the tendon, and the complex interactions among them. This review is aimed at providing an overview of recent advances in the identification and characterization of tendon stem/progenitor cells (TSPCs) and their interactions with ECM and mechanical loading. In addition, the effects of such interactions on the maintenance of tendon homeostasis and the initiation of tendon pathological conditions are discussed. Moreover, the challenges in further investigations of TSPC mechanobiology *in vitro* and *in vivo* are outlined. Finally, future research efforts are suggested, which include using specific gene knockout models and single-cell transcription profiling to enable a broad and deep understanding of the physiology and pathophysiology of tendons.

## 1. Introduction

Tendons are specialized tissues that enable joint movements by transmitting muscular forces from muscle to bone. They are relatively hypocellular tissues that are composed of an extracellular matrix (ECM), predominantly of collagen [1, 2], which is organized in a hierarchical manner. The collagen molecules assemble into fibrils that form fibers, fibers form fascicles, and bundles of fascicles form the fascicular matrix (FM). Endotenon, also known as the interfascicular matrix (IFM), occupies the space between fascicle bundles and is covered by epitenon and another layer of paratenon forming the whole tendon unit [1, 3]. Additionally, tendon contains two major types of cells, tenocytes and tendon stem/progenitor cells (TSPCs). Under normal conditions, tenocytes are responsible for maintaining tendon homeostasis, whereas TSPCs replenish tendon cells by undergoing self-renewal and differentiation [4, 5]. Tendon also contains other cell types such as endothelial cells, synovial cells of the tendon sheaths, and chondrocytes at the pressure and insertion sites in smaller amounts [3, 6].

The first study that isolated and characterized TSPCs indicates that these cell populations reside within the tendon proper (midsubstance) that comprises FM and IFM [4]. These cells are not strictly classified as “stem” cells, since they display heterogeneity in their biological properties. Instead, they were classified as “stem/progenitor” cells considering the possibility of inclusion of progenitor cells, which are destined to undergo differentiation towards a specific lineage. Indeed, while TSPCs possess multidifferentiation potential, they may contain progenitors that may specifically differentiate into tenocytes. Although TSPCs have been isolated and identified more than a decade ago, the lack of specific markers poses a challenge to study them further. In addition, distinctive populations of TSPCs have been identified from locations other than the tendon proper such as peritenon [7–9], but their functions are yet to be defined.

The hierarchical tendon structure is well optimized for its specific functions. Mechanical loads placed on the tendons are transformed into biochemical signals to tendon cells, which respond appropriately to regulate the metabolism of tendon and its structural properties [10, 11]. However, mechanical overloading may cause tendon injury, which is a common clinical problem affecting the quality of life for millions [12, 13]. Once tendon injury occurs, a successive natural healing process is thought to take place in three phases: inflammation (infiltration of inflammatory cells), proliferation (formation of new cells), and remodeling of ECM (change in the structure and form of tendon matrix) [14].

There are two categories of tendon injury: acute and chronic. Acute tendon injury, either partial or complete tear, results from a sudden tendon rupture that may be spontaneous or caused by direct trauma. Chronic tendon injury, commonly referred to as tendinopathy, is generally thought to result from repetitive mechanical overloading on the tendon, genetic predisposition, and age-related degeneration [15–17]. While pain and disability are the clinical indicators, the pathological features of tendinopathy include changes in the extracellular matrix (ECM) with collagen disorganization, proteoglycan deposition, neovascularization, and calcification [18, 19]. Several mechanisms have been proposed for the pathogenesis of tendinopathy that include with or without inflammation-mediated changes in tendon [20–22].

Due to the hypocellularity and hypovascularity of the tendon, the natural healing ability of tendons is rather limited [23, 24]. Moreover, tendon healing results in the formation of scar tissues, manifested by disorganized collagen matrix, increased proteoglycan and glycosaminoglycan content, and increased noncollagenous ECM [25–27]. Despite years of research, restoration of damaged tendon tissues to normal structure and function remains a great challenge in sports medicine and orthopaedic surgery. In particular, tendinopathic tendons respond poorly to current treatments including NSAIDS, corticosteroid and PRP injections, exercise-based physical therapy, and surgery [28]. Although heavy slow resistance (HSR) training reduces pain and improves collagen fibril morphology in a small number of patients [29], the efficacy of HSR training remains to be verified with large randomized controlled trials. By and large, current therapeutic strategies are palliative due to the limited understanding of the cellular and molecular mechanisms of tendinopathy. The development of new effective treatment options needs an in-depth understanding of basic tendon biology and, in particular, the function of tendon cells and their interactions with ECM in tendon.

Moreover, tendon is a mechanoresponsive tissue. Therefore, tendon homeostasis is maintained not only by the cells and ECM, but also by the mechanical loads placed on the tendon. TSPCs are responsive to mechanical loading, and some findings suggest that TSPCs are likely responsible for the development of degenerative tendinopathy by virtue of their multidifferentiation potential to nontenocyte phenotypes under excessive mechanical loading conditions [30–33]. Considering the emerging role of TSPCs in tendon homeostasis and in the development of tendon's pathological conditions, and their potential applications in tissue engineering of injured tendons, a deeper understanding of the interactions between TSPCs, ECM, and mechanical loading is essential. In this review, we discuss the efforts to identify and characterize TSPCs with regard to their locations in tendon. We also provide an overview of the interactions between TSPCs, ECM, and mechanical loading that may be important advances in tendon biology and pathology. Finally, we discuss the challenges in understanding TSPC biology and provide our perspectives on future research directions.

## 2. Tendon Cells

The cell populations in tendon are heterogeneous, and they are identified based on their anatomical locations such as FM, IFM, and paratenon, as well as the perivascular area close to paratenon in and around the tendon [34]. Still, there is little understanding of the phenotypical differences between these cell populations and specific markers to discriminate between them. The primary cell type in tendon is tenocytes, which are elongated fibroblast-like cells with spindle-shaped nuclei that are found mainly in FM [35]. Commonly used markers for tenocytes are collagen types I and III and tenomodulin (TNMD) [36]. It should be noted, however, that the term tenocyte in literature can be somewhat “arbitrary,” meaning that some so-called tenocytes are likely stem/progenitor cells.

Until the discovery of TSPCs, tenocytes were thought to be the only major cell type in tendon. The quest for the presence of adult stem cells in tendons began with two previous observations: (a) human and mouse tendons develop fibrocartilage and ossification in response to injury and (b) tendon-derived immortalized cell lines and human tendon-derived “fibroblasts” possess multidifferentiation capabilities *in vitro* [24, 37, 38]. Before long, TSPCs were first identified in humans and mice in 2007 [4]. The TSPCs isolated from the tendon proper with stem cell characteristics of clonogenicity, multipotency, and self-renewal could regenerate tendon-like tissues after *in vitro* expansion and *in vivo* transplantation [4]. In addition, an ECM-rich niche composed of biglycan and fibromodulin controls the self-renewal and differentiation of TSPCs [4]. Shortly, two other groups isolated and identified this unique stem cell population from tendons of rabbits and rats and characterized them extensively [5, 39]. In these studies, TSPC colonies exhibit large variations in cell proliferation and differentiation possibly due to differences in species, tissue origin, and initial seeding density in culture. The shape of TSPCs also varies between species, tissue origin, cell passages, and confluence of the culture [40]. Additionally, the success of obtaining a large pool of TSPCs depends on the age of the animal/individual; aged tendon tissues are depleted of at least 70% TSPCs, they proliferate much slower than young TSPCs, and they have much lower expression of stem cell markers [41].

TSPCs possess distinct properties compared to resident tenocytes. They differ from tenocytes in many aspects such as shape, proliferation and differentiation potential, and expression of stem cell-specific markers [5]. Rabbit TSPCs are more cobblestone-shaped with large nuclei, while tenocytes are more elongated, fibroblast-like with small nuclei in culture. Overall, TSPCs also proliferate much faster than tenocytes in culture [5]. Moreover, the capacity of multidifferentiation potency allows TSPCs to differentiate into tenocytes as well as nontenocytes, including adipocytes, chondrocytes, and osteocytes [4, 5, 39]. While both express common tendon-related markers including collagen type I, collagen type III, tenascin C, and TNMD, TSPCs *in vitro* express stem cell markers such as Oct-4, SSEA-1/4 and nucleostemin, while tenocytes exhibit a minimal expression of these markers [5].

TSPCs and bone marrow mesenchymal stem cells (BMSCs) share many of the same markers, yet the expression pattern is not identical between humans, mice, and rats [4, 41]. For example, human and mouse TSPCs lack CD18, but it is expressed in human BMSCs, and human and rat TSPCs do not express CD106, while it is expressed in human and mouse BMSCs [4, 39, 42, 43]. Also, over 60% of mouse TSPCs express CD90.2 whereas mouse BMSCs lack the expression [4]. Compared to mouse BMSCs, mouse TSPCs express higher mRNA levels of scleraxis (Scx), Comp, SOX-9, and Runx2. Human TSPCs also express higher levels of TNMD than human BMSCs [4]. Moreover, rat TSPCs have higher mRNA expression of tenogenic, adipogenic, and osteogenic markers compared to rat BMSCs at basal level [44]. These differences between species could suggest that TSPCs and BMSCs represent different developmental stages of a common MSC predecessor. Finally, since TSPCs tend to differentiate into tendon-specific cells (tenocytes) compared to BMSCs, whereas BMSCs tend to differentiate towards osteogenic lineage [4, 45], TSPCs may be ideal cells for tissue engineering of injured tendons.

## 3. Locations of TSPCs

**Figure 1:**
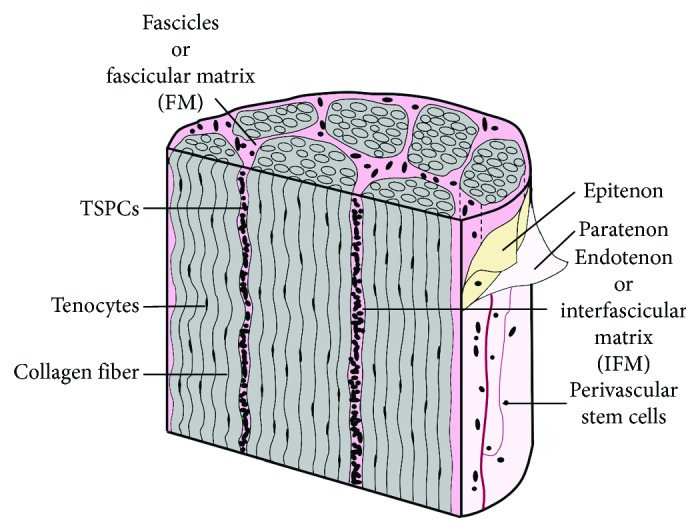
A simplified model of tendon structure adapted from Speisz et al. [47] showing fascicular matrix (FM), interfascicular matrix (IFM), and paratenon. The elongated tenocytes are located in FM in between fibers. The TSPCs from the tendon proper are presumably located in IFM; its exact location is yet to be determined, however.

The exact location of TSPCs in tendon is unclear. The IFM is a suggested location based on several observations. First of all, in the pioneering studies, TSPCs were isolated and characterized from the tendon proper after stripping off the tendon sheath and surrounding paratenon possibly to exclude vascular cells from the peritenon region [4, 5, 39], indicating IFM as a potential source of TSPCs. This speculation is strengthened by the observation that there are morphological and metabolic differences between IFM and FM. The IFM region is highly cellular and more vascular and has a fast turnover of noncollagenous matrix compared to FM, and the cells within IFM are round in shape compared to elongated tenocytes in FM (Figure 1) [46, 47]. However, tendon healing is thought to result from cells originating from multiple locations [48]. Therefore, it is possible that TSPCs may exist within each region in tendon and they may differ from one region to another, in terms of origins of progenitors, numbers of progenitor cells, and differentiation potentials. In fact, TSPCs have been isolated from peritenon/perivascular sources and their stem cell properties, such as clonogenicity, multipotency, and surface marker expression, have been determined and compared with TSPCs from the tendon proper (Table 1).

The perivascular area is an important source of stem/progenitor cells in tendon. Cells in intact human supraspinatus tendon biopsies and perivascular cells isolated from the microvessels of the same biopsies have been characterized. The results suggest that the perivascular region is a source of tendon precursor cells [7]. These cells express classical stem cell markers musashi-1, nestin, prominin-1/CD133, CD29, and CD44 as well as tendon-specific markers Scx and Smad 8. They also retain stem cell characteristics in culture. Later on, another study characterized TSPCs from the peritenon and tendon proper of mouse Achilles tendons [8]. Cells derived from the peritenon form less stem/progenitor cell colonies relative to those from the tendon proper. Analysis of surface markers for TSPCs from both regions indicated that they are Sca1^+^ (stem cell marker), CD90^+^, and CD44^+^ (fibroblast markers) (Table 1).

Progenitors from both the tendon proper and the peritenon demonstrate a low percentage of cells positive for leukocytic, hematopoietic, and perivascular markers CD18, CD34, and CD133, indicative of subpopulations of progenitor cells with stem cell properties, fibroblast features, and little contribution from leukocytic, hematopoietic, or perivascular sources. The marker profile of TSPCs isolated from the tendon proper is consistent with that described by Bi et al. [4]. Tendon proper stem/progenitor cells express high levels of TNMD and Scx, indicative of enrichment of stem/progenitor cells of a tendon origin. In contrast, cells of the peritenon demonstrate relative increases in the expression of vascular (endomucin) and pericyte (CD133) markers relative to cells from the tendon proper. However, cells from both regions were able to form primitive tendon constructs when seeded within a fibrin gel. These tendon constructs displayed tendon-like characteristics such as the expression of collagen type I and TNMD and formation of collagen fibril and fiber along the long axis. One particular distinction noted between the progenitors from the two sources was that when these cells were grown in osteogenic media, only progenitors from the tendon proper deposited calcium within the cell layer. This feature may provide an explanation for the calcification and ossification, which is a typical feature of tendinopathy in the tendon proper. Recently, transcriptome profiles of isolated murine Achilles tendon proper- and peritenon-derived progenitor cells were carried out [49]. It was found that progenitor cells from the tendon proper differ from peritenon progenitor cells in the differential expression of genes, including Scx, Mohawk, Thbs4, and Wnt10*α*. The distinct types of TSPCs within the tendon proper and the peritenon may differentially contribute to intrinsic (tendon proper) and extrinsic (epitenon and paratenon) tendon repair mechanisms. The intrinsic repair may require those progenitor cells that predominantly express tendon markers, while extrinsic repair may involve those stem cells recruited from the perivascular area.

To understand the location of tendon stem/progenitor cells in tendons and their role in tendon repair, *in vivo* identity of TSPCs and their role in tendon healing have been investigated in rats using the IdU label-retaining method [9]. The results showed that label-retaining cells (LRCs) could be identified at the tendon proper, peritenon, and tendon-bone junction. Most of the TSPCs isolated from the tendon proper were LRCs suggesting that LRCs were likely to be TSPCs isolated from tendon tissue. Most of the LRCs were found to be embedded between parallel collagen fibers; however, some LRCs were also found at the perivascular region at the peritenon, and these LRCs expressed CD146. Isolated TSPCs also expressed CD146 initially, but lost its expression during the *in vitro* expansion, although they still expressed Nanog, Oct-4, SOX-2, and nucleostemin. In the tendon injury model with a window defect, the LRCs migrated, proliferated, and activated for tenogenesis in the wound [9]. In another study, a subpopulation of cells exhibiting stem characteristics of clonogenicity, multipotency, and self-renewal capacity putatively of perivascular origin that reside within rat peritenon has been identified [50]. These cells expressed markers P75 (neurotrophin receptor), vimentin, SOX-10, and Snail consistent with neural crest stem cells (NCSCs). In the event of tendon injury, these perivascular cells may migrate from the vessels to interstitial space, and produce collagenous and noncollagenous proteins to repair damaged ECM [51].

The molecular profiling of individual cells derived from tendon identified a distinct subpopulation of nestin^+^ cells that express stem cell markers (CD146, CD105, etc.) and tenolineage markers (Col I, tenascin C, etc.) that are likely to be TSPCs [52]. Nestin is a type IV filament protein expressed in a variety of adult stem/progenitor cell populations that is required for the proper self-renewal [53–55]. Analysis of phenotypic differences between nestin^+^ TSPCs isolated *in vitro* from the tendon proper of human Achilles tendon shows a better tenogenic potential and self-renewing capacity and larger collagen fibril diameter than nestin^−^ TSPCs (Table 1). The nestin expression seems to be essential for the tenogenesis of TSPCs since the expression of nestin led to a strong induction of Scx and Mkx and tendon-related marker genes elastin and collagen type I and XIV. However, both nestin^+^ and nestin^−^ TSPCs display a similar propensity to differentiate into osteocytes, adipocytes, and chondrocytes and have similar proliferation potential. Nestin knockdown significantly reduces colony forming capacity and causes the loss of the typical shape of TSPCs. Nestin knockdown also impairs tendon repair and regeneration in a rat model of patellar tendon defect. Collectively, these data show that nestin could function as a marker for TSPCs. This is further strengthened by a previous study showing high levels of nestin expression in TSPCs isolated from human Achilles tendon [56]. Taken together, these studies identify sources for TSPCs including the tendon proper (midsubstance) and peritenon that may contribute towards tendon tissue maintenance, healing, or repair.

## 4. Interactions of TSPCs with ECM and Mechanical Loading

Stem cells cannot function without the signals from their niche. The various niche factors for stem cells include ECM and mechanical stress, as well as oxygen tension, growth factors, and cytokines [4, 40, 57, 58]. Considering the surrounding rich ECM, the main niche signals that TSPCs receive may be from those ECM components such as biglycan and fibromodulin [4]. The ECM microenvironment likely plays an important role in TSPC fate that ultimately affects tendon maintenance and repair when injury occurs to the tendon [4]. Alteration of the ECM may lead to tendon pathological conditions, but whether this altered composition of ECM will directly affect the fate of TSPCs remains rather unexplored.

In addition to rich collagen, tendon ECM contains small amounts of proteoglycans (PGs) [2, 35]. Small leucine-rich proteins (SLRPs) are the most abundant PGs present in tendon and act as the crucial components of ECM, as well as function as an organizer for collagen fibril assembly and regulators of ECM turnover [59, 60]. Decorin and biglycan are the main SLRPs in tendon. SLRPs such as fibromodulin and lumican are also present in tendon. The tendons of decorin/biglycan/fibromodulin-deficient animals are mechanically inferior to normal tendons of wild-type mice [61, 62], and the collagen fibers within the tendon become disorganized in the absence of biglycan and fibromodulin [4]. Tendon integrity is impaired in lumican and fibromodulin-deficient mice [63]. Alteration of the ECM composition changes the structure of the TSPC niche consequently affecting the fate of TSPCs, which leads to tendon malformation and ossification [4]. Biglycan and fibromodulin are two critical SLRPs that control the fate of TSPCs. This may be mediated in part by modulating bone morphogenic protein (BMP) activity. TSPCs from biglycan and fibromodulin double-knockout mice proliferate faster, form larger colonies, and form bone-like tissues in addition to tendon-like tissues compared to those from wild type (WT) mice which form only tendon-like tissues [4]. The increased sensitivity of TSPCs to BMP-2 in the absence of biglycan and fibromodulin could be a mechanism for altering the fate of TSPCs. The expression of tendon markers Scx and collagen type I is decreased in TSPCs from these knockout mice compared to cells from WT mice. Therefore, the integrity of ECM is important in maintaining the stemness of TSPCs, and the precise regulation of the tenogenic differentiation of TSPCs is essential for the positive outcome of stem cell-based therapy for injured tendons.

In tendon, cell-ECM interactions maintain tissue homeostasis by generating cell signals that affect cell proliferation, differentiation, migration, and adhesion [35]. On the other hand, the ECM plays an important role in disease progression. The tendon ECM is enriched in growth factors and cytokines, and the ECM plays a major role in regulating the local availability of growth factors at a cellular level [64]. The changes of the structure and composition of ECM may disturb the local release of growth factors and cytokines as well as the modulation of cell shape and signaling cascade affecting the cell fate. Aberrant ECM changes including calcification, ossification, and lipid and proteoglycan accumulation are evident in human tendinopathy samples [65, 66]. The aberrant differentiation of TSPCs to nontenocytes (adipocytes, chondrocytes, and osteoblasts), which produce nontendinous tissues, is suggested as a possible mechanism in the development of tendinopathy due to mechanical overloading placed on the tendon [30, 32, 33].

An engineered tendon matrix (ETM) from decellularized tendon tissues stimulates rabbit TSPC proliferation and better preserves stemness compared to plastic culture surfaces commonly used in culture, and implantation of ETM-TSPC composite promotes tendon-like tissue formation [67]. The ECM components and/or growth factors may contribute these properties to TSPCs, which are important in tissue engineering applications of such composites in injured tendon repair. A similar study using decellularized collagenous matrix from three different tissues (tendon, bone, and dermis) showed that tendon-derived decellularized matrix promotes the tendinous phenotype in human TSPCs and inhibits their osteogenesis, even under osteogenic induction conditions, although all the three matrices support cell adhesion and proliferation [68]. The bone-derived decellularized matrix robustly induces osteogenic differentiation of TSPCs, whereas the dermal skin-derived collagen matrix induces only an intermediate level of osteogenesis. The differential cellular response could be attributed to the differences in the structure and topography but otherwise similar bioactivity of the matrices. The cell shape and alignment also differed in the three matrices; cells adopt an elongated shape and align on the tendon matrix, but not on the dermis matrix. This shows that besides the composition, ECM topographical cues are important in regulating the stem cell fate. This is supported by yet another study, which indicated that the culture of human TSPCs in an aligned nanofiber scaffold promotes tenogenic commitment, but in a random scaffold enhances osteogenic differentiation [42]. Compared to embryonic stem cell-mesenchymal stromal cells, TSPCs combined with the decellularized matrix also show more improvement in the structural and biomechanical properties of regenerated tendons *in vivo* [69]. In short, ECM components provide niche signals to TSPCs and play a significant role in deciding their fate depending on the changes in ECM composition and topographical cues.

The precise function of TSPCs *in vivo* is not well defined yet, and comparison studies with tenocytes are rare. Tendons have poor regenerative capacity as demonstrated by the inferior quality of tissues following injury or chronic degeneration [70, 71]. Therefore, it is conceivable that TSPCs alone may not be able to functionally restore the damaged tissues, although TSPCs promote functional repair of tendon tissues [72–77].

It is well known that mechanical loads play a major role in tendon development, homeostasis, pathology, and injury healing. These forces are translated into biochemical signals by molecules possessing mechanotransduction capabilities which activate and control key cellular processes of tendon [11, 78]. Normal mechanical loads are essential for appropriate tendon development and maintenance, because such loads like moderate loading patterns induce cellular anabolic adaptation of tendon [78–80]. On the other hand, abnormal mechanical loads cause pathological conditions (e.g. tendinopathy) in tendon by inducing dominant catabolic responses in tendon cells [14, 81–85]. Tendon cells respond to mechanical loads and modulate ECM via various mechanisms/pathways which have been extensively investigated using both *in vitro* and *in vivo* loading models (e.g., [86–88]).

TSPCs are capable of altering the tendon ECM in response to modifications of the loading environments. Also, the multidifferentiation potential of TSPCs allow them to differentially respond to altering mechanical loads. For example, an *in vitro* study showed that a uniaxial cyclic mechanical stretching of patellar and Achilles TSPCs from mice at moderate levels (4% elongation, 0.5 Hz for 12 hrs) increases proliferation and collagen type I gene expression without affecting the gene expressions of PPAR*γ* (a marker for adipocytes), collagen type II, SOX-9 (markers for chondrocytes), and Runx2 (a marker osteocytes) [30]. Similarly, mechanical loading in the form of moderate treadmill running in mice increases the proliferation of TSPCs and TSPC-related cellular production of collagen [57].

However, mechanical stretching of mouse TSPCs at an excessive level (8% elongation) increases the gene expression of PPAR*γ*, collagen type II, SOX-9, and Runx2 [30]. A further study showed that a 4% stretching of TSPCs increases the expression of tenocyte-related genes (collagen I and TNMD) while 8% stretching increased the expression of both tenocyte and non-tenocyte-related genes (LPL, SOX-9, and Runx2) in TSPCs but not in tenocytes [33]. The increase in both tenocyte and nontenocyte-related gene expression under 8% stretching may be due to the fact that the TSPC population is heterogeneous, meaning that individual TSPC may have different levels of threshold in response to mechanical loading and as a result, they respond differently in their gene expression. Similar results were obtained when the study was performed *in vivo* using moderate and intensive treadmill running to apply low and excess mechanical loading, respectively [33]. Although the results from cellular and tissue levels are presented only at gene expression levels, it may indicate that mechanical overloading may prime TSPCs to undergo nontenocyte differentiation. Further studies are warranted to link the nontenogenic differentiation of TSPCs and degenerative changes in tendinopathy.

Also, in another study, mechanical stretching at 4% and 8% (0.5 Hz for 4 hrs) increased BMP-2 expression at gene and protein levels in rat TSPCs [31]. BMP-2 increased osteogenic differentiation of TSPCs as indicated by ALP activity and calcium nodule formation. The observation of osteogenic differentiation at 4% is in contrast with the previous findings [30, 33], possibly due to the differences in the stretching regimen, species difference, and culture conditions. Involvement of BMP-2 in the pathogenesis of tendinopathy has been suggested previously based on the reported observations that chondrocyte phenotype and ectopic ossification are present in calcifying tendinopathy and based on the expression of BMP-2 protein at those sites [89–92]. Moreover, the addition of BMP-2 to human TSPCs in culture decreased cell proliferation and induced osteogenic differentiation [93]. Higher BMP-2 receptor expression and BMP-2-induced osteogenic differentiation of rat TSPCs compared to BMSCs have been reported [94]. Taken together, these studies indicate that the activation of BMP-2 expression in TSPCs during tendon overuse might provide a possible explanation for ectopic calcification in calcifying tendinopathy. Overall, the data indicate that moderate mechanical loads are beneficial for maintaining tendon homeostasis, but mechanical overloading may contribute to degenerative changes in tendon, with TSPCs playing a major role.

## 5. Conclusion

TSPCs have been identified in both the tendon proper and peritenon in tendon that may have both vascular and nonvascular origins. However, how TSPCs from different locations contribute to tendon maintenance, repair, and tendinopathy remains to be better understood. This is indeed a challenging task now considering the lack of definitive genetic lineage tracing and specific markers for TSPC identity, functions, and biological characterization. To this end, advanced studies using state-of-the-art novel approaches, including genetic models, genetic lineage tracing, and single-cell transcription profiling to identify the heterogeneity of TSPCs, need to be conducted.

TSPCs reside in tendons and are constantly subjected to mechanical loading due to the fact that tendons like Achilles and patellar are load-bearing tissues. Consequently, the function of TSPCs is regulated by ECM composition, organization, and mechanical loads. Therefore, further investigations are necessary to understand the crosstalk among TSPCs, ECM, and mechanical loads, and also those signaling pathways involved, so that tendon physiology and pathophysiology can be better understood. The findings from these investigations will surely aid in devising new yet effective tissue engineering approaches to regenerate injured tendons and also developing novel treatment strategies to manage tendinopathy, a prevalent tendon disorder commonly seen in both athletic and general populations.

## Figures and Tables

**Table 1:** Sources and properties of TSPCs.

Sources	Properties	Markers	Reference
Tendon proper/midsubstance	(i) Typical stem cell characteristics (ii) Multipotent (iii) Distinct from BMSCs	Scleraxis, TNMD, collagen type I, tenascin, Sca1, CD90.2, CD44, CD146, Oct-4, SSEA-4, nucleostemin	Bi et al., 2007 [4]; Zhang and Wang, 2010 [5]; Rui et al., 2010 [39]
	(i) Typical stem cell characteristics (ii) Form tendon constructs that express collagen type I and TNMD (iii) Deposit calcium in osteogenic medium (iv) Potential nonvascular origin	Sca1, CD90, CD44, CD19, CD34, CD13, musashi-1, TNMD, scleraxis	Mienaltowski et al., 2013 [8]
	(i) Typical stem cell characteristics (ii) Potential nonvascular origin	CD146, Oct-4, nanog, SOX-2, nucleostemin	Tan et al., 2013 [9]
	(i) Typical stem cell characteristics (ii) Higher self-renewal and tenogenesis capacity, larger collagen fibril diameter compared to nestin-negative TSPCs	Nestin, CD146, CD90, CD44, CD105, CD51	Yin et al., 2016 [52]

Peritenon	(i) Express classical stem cell markers (ii) Retain stem cell characteristics in culture (iii) Potential vascular origin	Musashi-1, nestin, prominin-1/CD133, nestin, collagen types I and III, Smad8, CD29, CD44, scleraxis	Tempfer et al., 2009 [7]
	(i) Typical stem cell characteristics (ii) Display TSPC surface profile (iii) Form tendon constructs that express collagen type I and TNMD (iv) Potential vascular origin	Sca1, CD90, CD44, CD19, CD34, CD13, musashi-1	Mienaltowski et al., 2013 [8]
	(v) Both vascular and nonvascular sources	CD146, Oct-4, Nanog, SOX-2, nucleostemin	Tan et al., 2013 [9]
	(i) Typical stem cell characteristics (ii) Neural crest-like stem cells (iii) Potential vascular origin (iv) Involved in tendon repair	CD29, CD90, P75, vimentin, Snail, SOX-10	Xu et al., 2015 [50]

## Abbreviations

ALP: Alkaline phosphatase
BMP-2: Bone morphogenetic protein-2
BMSCs: Bone marrow mesenchymal stem cells
Col I: Collagen type I
Comp: Collagen oligomeric matrix protein
ECM: Extracellular matrix
ETM: Engineered tendon matrix
FM: Fascicular matrix
GAG: Glycosaminoglycans
HSR: Heavy slow resistance
IFM: Interfascicular matrix
LPL: Lipoprotein lipase
LRC: Label-retaining cells
NSAIDs: Nonsteroidal anti-inflammatory drugs
NCSCs: Neuronal crest stem cells
Oct-4: Octamer-binding transcription factor 4
PGs: Proteoglycans
PPAR*γ*: Peroxisome proliferator-activated receptor *γ*
PRP: Platelet-rich plasma
Runx2: Runt-related transcription factor 2
Scx: Scleraxis
SLRPs: Small leucine-rich proteins
Smad8: Mothers against decapentaplegic homolog 8
SOX-9: Sex-determining region SRY
SSEA-1/4: Stage-specific embryonic antigen 1/4
Thbs4: Thrombospondin 4
TNMD: Tenomodulin
TSPCs: Tendon stem/progenitor cells.
